# *PCSK9-*D374Y Suppresses Hepatocyte Migration through Downregulating Free Cholesterol Efflux Rate and Activity of Extracellular Signal-Regulated Kinase

**DOI:** 10.1155/2023/6985808

**Published:** 2023-01-09

**Authors:** Lei Huang, Ying Cheng, Yulian Mu, Kui Li

**Affiliations:** ^1^Shenzhen Branch, Guangdong Laboratory of Lingnan Modern Agriculture, Key Laboratory of Livestock and Poultry Multi-omics of MARA, Agricultural Genomics Institute at Shenzhen, Chinese Academy of Agricultural Sciences, Shenzhen 518120, China; ^2^State Key Laboratory of Animal Nutrition, Institute of Animal Sciences, Chinese Academy of Agricultural Sciences, 2 Yuanmingyuan West Road, Haidian District, Beijing 100193, China

## Abstract

Proprotein convertase subtilisin/kexin type 9 can mediate the intracellular lysosomal degradation of the low-density lipoprotein receptor protein in hepatocytes and decrease the liver's ability to scavenge low-density lipoprotein cholesterol from circulation, resulting in high levels of cholesterol in the circulatory system. Current studies have primarily focused on the relationship between *PCSK9* and blood lipid metabolism; however, the biological function of *PCSK9* in hepatocytes is rarely addressed. In this study, we evaluate its effects in the human hepatoma carcinoma cell line HepG2, including proliferation, migration, and free cholesterol transport. *PCSK9-*D374Y is a gain-of-function mutation that does not affect proliferation but significantly suppresses the migration and cholesterol efflux capacity of these cells. The suppression of the transmembrane outflow of intracellular-free cholesterol regulates small G proteins and the suppression of extracellular signal-regulated kinase. In summary, *PCSK9-*D374Y affects hepatocyte features, including their migration and free cholesterol transport capabilities.

## 1. Introduction

The proprotein convertase subtilisin-like/kexin type 9 (*PCSK9*) gene is one of the major genes associated with familial hypercholesterolemia [1, 2]. It regulates plasma LDL-C (low-density lipoprotein cholesterol) levels by posttranslational regulation of the LDLR (low-density lipoprotein receptor), according to the following: secreted protein binds LDLR on the hepatocyte membrane, mediates its degradation via intracellular lysosomal and the ubiquitylation pathway, and blocks the recycling of LDLR on the cell membrane, which increases cholesterol levels in circulation [3–5]. Current studies have primarily focused on the relationship of *PCSK9* with lipid balance and cholesterol metabolism, especially plasma LDL-C levels and blood lipid metabolism.

*PCSK9* is primarily expressed in cells with regeneration and differentiation capabilities such as hepatocytes, macrophage, and smooth muscle cells [6–8]. It can affect the biological characteristics of macrophages through autocrine or paracrine modes of action [6]. The liver is typically the primary target organ for this action [9]. Hepatic transcriptome and proteome analyses have revealed that *PCSK9* overexpression can affect steroid synthesis and the expressions of genes involved in stress response and immune pathways, suggesting that *PCSK9* also participates in biological processes other than cholesterol metabolism in hepatocytes [9–12]. Additionally, *PCSK9* is associated with many human diseases such as liver disease, kidney disease, and diabetes, [13–15]. Regulation of *PCSK9* activity can impact patients with liver disease [14] such as hepatic steatosis. A liver function test has been done in patients with familial hypercholesterolemia taking *PCSK9* inhibitors. The risk factors of liver injury like alanine transaminase and aspartate transaminase are increased after 6 months of treatment [16]. In addition, in a mouse model with liver injury, the loss of *PCSK9* activity can severely suppress the regeneration and repair of the liver [17]. Therefore, *PCSK9* can have many potential targets, which should be accounted for during drug research and development and in clinical trials to avoid any side effects during drug administration.

The *PCSK9*-D374Y mutant has been identified in a Utah family [18]. The binding ability of the D374Y mutant to LDLR on the hepatocyte membrane is ten times that of the wild type [19]. This greatly weakens the liver's affinity for eliminating LDL-C. Since *PCSK9* can increase serum LDL-C, it has become a subject of focus in the research and development of novel lipid-lowering drug targets. However, few studies have explored the relationship between *PCSK9* and the biological function of hepatocytes. In this study, we investigate cholesterol efflux, cell proliferation, and migration in hepatocytes. We use the *PCSK9*-D374Y mutant, which shows stable high expression in hepatocytes and mimics the microenvironment of high protein *in vitro*, to explore its effect on the biological functions of hepatocytes.

## 2. Material and Methods

### 2.1. Construction of PCSK9 Overexpression Vectors

The *PCSK9* overexpression vector (either wild type or the D374Y mutant) was constructed using pcDNA3.1(+) as the backbone vector containing the neomycin gene. The plasmid contains a MAR insulator (*G. gallus* lysozyme gene 5′ matrix attachment region subfragment B-1-H1), which is immediately followed by the human APOE promoter and the cDNA sequence of *PCSK9* (Figure S1). The sequence of the D374Y mutant is directly synthesized (Supplementary Information). The synthesis and construction of the vector were completed by Genewiz, Suzhou, China. These vectors were named pcDNA3.1-*PCSK9* and pcDNA3.1-*PCSK9*-D374Y. The empty vector of pcDNA3.1 was used as negative control (NC).

### 2.2. Establishment of HepG2 Cells Stably Overexpressing PCSK9 and *PCSK9*-D374Y

The human liver cancer cell line HepG2 was purchased from Peking Union Medical College Cell Center. Cells were cultured in Dulbecco's modified eagle medium (DMEM, Thermo Fisher Scientific, USA) containing 10% fetal bovine serum (FBS, Thermo Fisher Scientific). HepG2 cells were plated in six-well plates (Corning, USA) at a density of 1 × 10^6^ cells/well. After 48 h, the plasmid was linearized and transfected using FuGENE HD Transfection Reagent (Promega, USA). Cell death was monitored 12 h after transfection; the medium was changed immediately if the mortality rate is >10% or changed 24 h after transfection if the mortality rate is <10%. G418 was added at 400 *μ*g/ml to screen positive cells and cultured cells for another 10-14 days until cell death occurred, which indicated a high degree of enrichment of positive cells. Pooled positive cells were maintained with 200 *μ*g/ml G418.

### 2.3. Cholesterol Efflux Assay

Cells were plated in six-well plates at a density of 2 − 3 × 10^6^/well. Twelve hours later, they were cultured in a medium containing 2.5% BSA and 10 *μ*M of 22-NBD-cholesterol (Thermo Fisher Scientific). Cells were incubated for 3 h and washed twice with PBS, after which the medium was replaced by serum-free and phenol red-free medium with HDL (10 *μ*g/ml) (total HDL extracted from the plasma of healthy volunteers, Cardiovascular Research Institute, Peking University Health Science Center) to initiate the cholesterol efflux assay [20, 21]. The medium was removed after 2 h and the cells were harvested and resuspended in PBS. The intracellular fluorescence intensity (FI) was measured via flow cytometry to identify the 22-NBD-cholesterol content. The cholesterol efflux rate was calculated according to the following formula:

(1) $$\begin{array}{c} \text{Percent efflux}=\frac{\text{Total fluorescent from the cells in the absence of HDL}-\text{total fluorescent from the cells in the presence of HDL}}{\text{Total fluorescent from the cells in the absence of HDL}}. \end{array}$$

### 2.4. Cell Proliferation Assay Using Cell Counting Kit-8 (CCK-8)

A CCK-8 reagent kit was purchased from Dojindo, Shanghai, China. The standard proliferation curve of the HepG2 cells was drawn as follows. Wild-type cells (HepG2 cells without any genetic modification) were grown to 90% confluency and collected and diluted at concentration gradients with 8-10 wells set for each concentration (2000, 4000, 6000, 8000, and 10000). The cells were then plated in a 96-well plate and cultured for 2-4 h. After the cells reached complete adherence, 10 *μ*l of CCK-8 reagent was added into each well for a total reaction volume of 110 *μ*l. After culturing cells for another 1-2 h, optical density (OD) measurement was performed using an optical densitometer (Thermo Fisher Scientific, EV60) when the medium color changes. Measurements in the blank wells were used as background values for normalization. The linear standard curve was drawn with the cell count on the *x*-axis and the OD value on the *y*-axis, and the standard curve was used to determine cell numbers under the same condition. Cell proliferation was detected after the standard curve was drawn. Each group has 4 × 10^3^ cells per well with ten technical duplications. OD measurements were performed after 24 h, 48 h, and 72 h of culture in separate wells. The number of cells was fitted according to the standard curve.

### 2.5. EdU Cell Proliferation Assay

The reagent kit for EdU cell proliferation assay was purchased from Guangzhou RiboBio Co., Ltd., Guangdong, China. The cell proliferation assay was performed according to the manufacturer's instructions. The experimental process included EdU labeling, cell fixation, Apollo dying, DNA dying, and image acquisition and analysis. Cells in the logarithmic phase were laid into 96-well plates with 4 × 10^3^ cells per well. After 24 h of culture, the cells were incubated with 50 *μ*M EdU for 2 h, after which they were fixed with 4% paraformaldehyde. 1× Apollo and 1× Hoechst 33342 dying reaction solutions are ready-to-use, according to the instructions. The nucleus-emitting red fluorescence indicated cells with DNA replication activity. All the nuclear cells were stained in blue by Hoechst 33342. Ten pictures of each group were randomly collected using a fluorescence microscope (Olympus, Tokyo, Japan, BX53). The proliferation rate of the cells was analyzed by GraphPad Prism software by calculating the ratio of number of red blood cells to the number of blue blood cells.

### 2.6. Cell Migration Assay

For scratch wound healing assays, HepG2 cells stably expressing *PCKS9-*D374Y or NC cells were plated in a six-well plate at 4 × 10^5^/ml, with triplicate wells in each group, in DMEM complete culture medium for 24 h. Cells were treated with 10 *μ*g/ml mitomycin C for 2 h to arrest cell division. Three parallel and even scratches were made along the plate bottom using a 10 *μ*l pipette tip. Floating cells were removed by rinsing them with a serum-free culture medium several times. The cells were cultured, and the scratch healing was monitored and photographed every 24 h. Photos of nine visual fields in each well were obtained to qualitatively compare the scratch areas. *In vitro* transwell migration assay was performed according to a previously described method [22]. HepG2 cells of 4 × 10^4^ were placed in the upper chamber of the transwell assembly (6.5 mm diameter inserts and 8.0 *μ*m pore size; Corning) in 100 *μ*l FBS-free DMEM basal medium. Meanwhile, 800 *μ*l of DMEM complete culture medium containing 10% FBS filled the lower compartments as a source of chemoattractant. The membrane was incubated at 37°C for 12 h and then stained with Hoechst 33342 (Beyotime Institute of Biotechnology, China). The number of migrating cells was determined by counting 10 random fields per well with a fluorescence microscope (Olympus, BX53), with three sets for each group.

### 2.7. GTPase Activity Assay

The G protein activity was determined by G-LISA assay, which has been described in the literature [22]. The activities of RHOA-, RAC1-, and CDC42-GTP were detected using G-LISA kits (Cytoskeleton, USA) according to the manufacturer's instructions. Plates with HepG2 cells cultured with serum-free medium for 6 h were placed on ice. The cells were washed with precooled PBS three times and allowed to stand for 1 min, after which 100 *μ*l of protein lysis buffer (M-PER mammalian protein extraction reagent, Thermo Fisher Scientific) (containing 1 mM of protease inhibitors, Thermo Fisher Scientific) was added to each well. Cells were collected and placed in 1.5 ml Eppendorf tubes for lysis on ice for 10 min. Next, 10 *μ*l of lysates was added into the wells of a 96-well plate, to which 290 *μ*l of reagents was added for protein analysis. The OD value was detected at 600 nm 1 min later, and the concentration value was calculated by subtracting the value in the blank control group.

### 2.8. Nuclear and Cytoplasmic Extraction

Nuclear and cytoplasmic extracts from HepG2 cells were obtained using a Nuclear and Cytoplasmic Extraction Kit (CWBio, CW0199, China).

### 2.9. Quantitative Real-Time PCR (qPCR)

To verify the effect of overexpression of *PCSK9*-D374Y, target gene expression was quantified by qPCR and normalized to GAPDH levels. Total RNA samples were treated with DNase I before reverse transcription using random priming and Superscript Reverse Transcriptase (Thermo fisher Scientific), according to the manufacturer's instructions. qPCR was performed using ABI StepOne (Thermo fisher Scientific) and PowerUp SYBR Green Master Mix (Thermo Fisher Scientific). Gene expression was evaluated using the ∆∆Ct method.

### 2.10. Western Blotting

Western blot analysis was performed according to previously published methods [23]. The cell samples were briefly lysed with mammalian protein lysis buffer (Thermo Fisher Scientific) containing a protease inhibitor mixture (Roche Applied Science, Switzerland). Twenty micrograms of total protein was separated by 10% (wt/vol) SDS-PAGE and transferred onto a nylon membrane (Merck Millipore, Germany). After being blocked with 2% (wt/vol) bovine serum albumin (Sigma-Aldrich, Saint Louis, MO 63103, USA), the antibodies were then used to probe the membrane. The following antibodies were used: histone H2A (CST, 12349, Danvers, Massachusetts, USA) 1 : 1000, LDLR (Millipore, MABS26) 1 : 1000, PCNA (CST, 13110) 1 : 1000, *PCSK9* (Circulex, CY-P1037, MBL, Japan) 1 : 1000-2000, *β*-actin (CST, 4970) 1 : 1000, GAPDH (CST, 5174) 1 : 1000, Akt (pan) (CST, 4691) 1 : 1000, phospho-Akt (Ser473) (CST, 4060) 1 : 1000, p44/42 MAPK (Erk1/2) (CST, 4695) 1 : 1000, and phospho-p44/42 MAPK (Erk1/2) (Thr202/Tyr204) (CST, 4370) 1 : 1000. The secondary antibodies used in the western blotting are as follows: anti-mouse IgG, HRP (CST, 7076) and anti-rabbit IgG, HRP (CST, 7074). The protein expression in the western blot analysis was measured by gray analysis software (ImageJ).

### 2.11. Enzyme-Linked Immunosorbent Assay (ELISA)

Secreted *PCSK9* in the HepG2 cell culture medium was detected using a Human *PCSK9* ELISA Kit (MBL, Japan), according to the manufacturer's instructions.

### 2.12. Flow Cytometry

The LDLR expression level on the membrane surface of HepG2 cells was determined by flow cytometry. The cultured HepG2 cells were collected and washed with ice-cold flow cytometry staining buffer (R&D Systems, Minnesota, USA) three times. Cell pellets were resuspended in blocking IgG solution (1 *μ*g IgG/10^6^ cells) for 15 min at 4°C to block nonspecific binding. Antibodies used include mouse anti-human LDLR monoclonal Alexa Fluor® 488 and mouse IgG1 isotype control Alexa Fluor® 488 (R&D Systems), while assays were performed according to the flow cytometry protocol for staining membrane-associated proteins (R&D Systems). The flow cytometric acquisition and data analysis were performed using a BD FACSCalibur flow cytometer and CellQuest software. Three independent flow cytometric experiments were performed.

### 2.13. Data Analysis

The experimental data were analyzed using GraphPad Prism software. Regular two-way ANOVA analysis was performed. Variance among different groups was analyzed using Sidak's multiple comparison test, and *P* value < 0.05 was considered statistically significant.

## 3. Results

### 3.1. Overexpression of *PCSK9*-D374Y in HepG2 Cells Decreases LDLR Levels

To explore the biological function of *PCSK9*-D374Y in hepatocytes, a *PCSK9-*D374Y overexpression vector is constructed, which is shown in Figure S1A. The results of the vector sequencing (not provided) and restriction enzyme digestion indicate that the pcDNA3.1-*PCSK9*-D374Y overexpression vector was successfully constructed (Figure S1B).

The transcription, expression, and secretion of *PCSK9* were confirmed in HepG2 cells. The mRNA and protein were both significantly upregulated in *PCSK9*-D374Y mutant overexpressed HepG2 cells (Figure S2A, S2B) (mRNA, *P* < 0.0001, *t*-test; protein, *P* = 0.023, *t*-test). Moreover, *PCSK9* secretion also significantly increased (Figure S2C) (*P* < 0.0001,  *t*-test).

**Figure 1:**
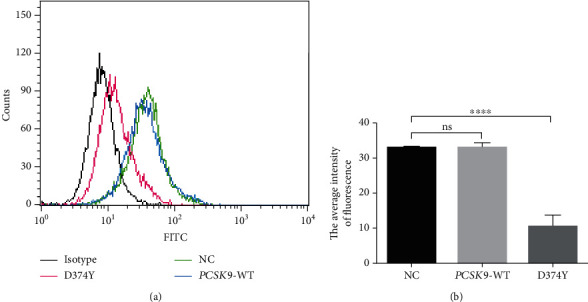
Effect of *PCSK9* overexpression on the level of LDLR in HepG2 cells. (a, b) Flow cytometry analysis of LDLR protein levels on the cellular membrane in HepG2 cells transfected with pcDNA3.1-*PCSK9*-D374Y, pcDNA3.1-*PCSK9*-WT, or pcDNA3.1 vector. Black line: cells incubated with isotype control antibody, red line: cells transfected with pcDNA3.1-*PCSK9*-D374Y, green line: cells transfected with pcDNA3.1, and blue line: cells transfected with pcDNA3.1-*PCSK9*-WT. A bar chart is shown in (b) to compare the mean FI from different groups from (a). At least three independent flow cytometry experiments were conducted with triplicate items. The bar chart is shown as mean with SEM. ns: not significant; ^∗∗∗∗^*P* < 0.001.

To validate the effect of the *PCSK9*-D374Y in decreasing LDLR levels in hepatocytes, the protein levels of LDLR were analyzed in HepG2 cells using flow cytometry. Overexpression of the *PCSK9*-D374Y mutant significantly decreased the level of LDLR in the cell membrane (Figures 1(a) and 1(b)) (*P* < 0.0001, *t*-test). These results indicated that overexpression of the *PCSK9*-D374Y had enzyme activity and can mediate LDLR degradation in HepG2 cells.

### 3.2. *PCSK9*-D374Y Suppresses the Cholesterol Efflux Rate of HepG2 Cells

**Figure 2:**
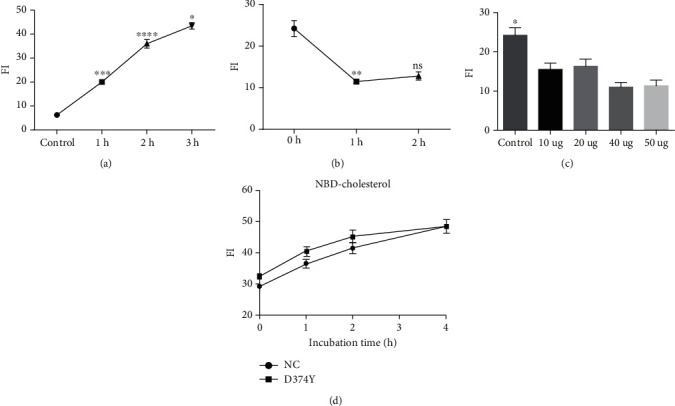
Cholesterol efflux assay in HepG2 cells. (a) Relationship between NBD-cholesterol incubation time and FI. (b) Relationship between HDL treatment time and cholesterol efflux. HDL concentration used in (a) and (b) is 10 *μ*g/ml. (c) Relationship between HDL concentration and cholesterol efflux. HDL treatment time is 2 h. Wild-type HepG2 cells are used for (a)–(c). (d) Effect of *PCSK9* overexpression on cholesterol labeling in HepG2 cells. NC: pcDNA3.1; D374Y: pcDNA3.1-*PCSK9*-D374Y; FI: fluorescence intensity. At least three independent experiments were conducted with triplicate items. Data is shown as mean with SEM. ns: not significant; ^∗^*P* < 0.05; ^∗∗^*P* < 0.01; ^∗∗∗^ or ^∗∗∗∗^*P* < 0.001.

Previous studies have shown that *PCSK9* reduces the LDLR level in hepatocytes and suppresses LDL-C uptake by hepatocytes, thus affecting cholesterol metabolism [24]. However, few studies have explored the relationship between *PCSK9* and cholesterol transport. We used NBD-cholesterol as a marker to identify the effects of high *PCSK9*-D374Y expression on cholesterol efflux in HepG2 cells. Wild-type HepG2 cells were used to test incubation time, efflux time, and the dosage of high-density lipoprotein (HDL). Results demonstrated that cholesterol labeling in HepG2 cells increased with incubation time (Figure 2(a)). HDL-mediated cholesterol efflux reaches a plateau at 2 h (Figure 2(b)) and is not dose-dependent on HDL (Figure 2(c)). However, high *PCSK9*-D374Y expression has no significant effect on cholesterol labeling in HepG2 cells compared with the control group (Figure 2(d)). HDL at a concentration of 10 *μ*g/ml can produce a remarkable cholesterol efflux effect, which can then be used for detection.

**Figure 3:**
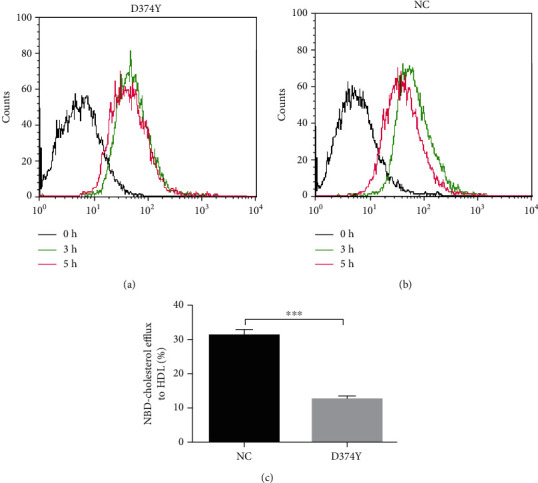
Effects of *PCSK9* overexpression on free cholesterol transport in HepG2 cells. (a–c) Effects of *PCSK9* overexpression on cholesterol transport in HepG2 cells. (a) and (b) show peaks in the flow cytometry chart, indicating deviations of the mean FI curve during cholesterol efflux. Black line: background FI in HepG2 cells, green line: results of incubation with NBD-cholesterol for 3 h (i.e., the starting time point of cholesterol efflux), and red line: results of HDL treatment for 2 h following 3 h of inoculation with fluorescence cholesterol. FI: fluorescence intensity. The entire assay lasted 5 h. FI indicates the content of fluorescent-labeled cholesterol inside cells. NC: pcDNA3.1; D374Y: pcDNA3.1-*PCSK9*-D374Y. (c) is the bar chart for cholesterol efflux rate. At least three independent flow cytometry experiments were conducted with triplicate items. The bar chart is shown as mean with SEM. ^∗∗∗^*P* < 0.001.

**Figure 4:**
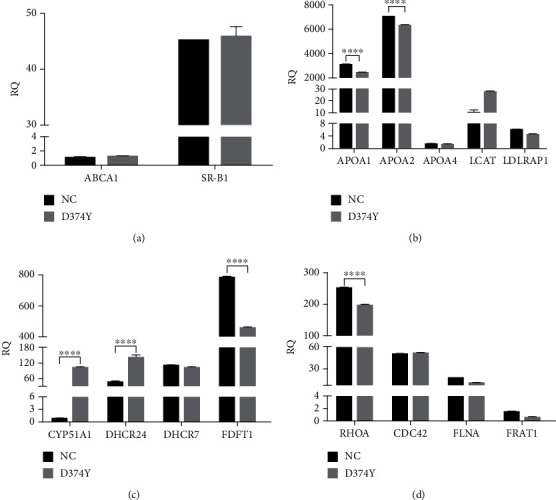
QPCR detection of genes related to cholesterol metabolism and cell migration. Expression of genes related to cholesterol efflux (a), cholesterol transport (b), cholesterol biogenesis (c), and cell migration (d). NC: pcDNA3.1; D374Y: pcDNA3.1-*PCSK9*-D374Y.

We next analyzed the cholesterol efflux rates in stable cell lines treated with NBD-cholesterol for 3 h after which the cholesterol efflux was mediated with 10 *μ*g/ml HDL for 2 h. Compared to the control, cells overexpressing *PCSK9*-D374Y show significantly inhibited cholesterol efflux (Figure 3) (*P* < 0.001, *t*-test), suggesting that *PCSK9*-D374Y could negatively affect free cholesterol transport in hepatocytes. The transcription of cholesterol efflux-related genes such as ABCA1 and SR-BI was not influenced by *PCSK9*-D374Y (Figure 4(a)). However, cholesterol transport-related genes such as APOA1 and APOA2 were significantly downregulated (Figure 4(b)) (*P* < 0.0001, *t*-test), while cholesterol biogenesis-related genes such as CYP51A1 and DHCR24 are significantly upregulated (Figure 4(c)) (*P* < 0.0001, *t*-test). These results imply that *PCSK9*-D374Y is indirectly involved in the inhibition of cholesterol efflux.

### 3.3. *PCSK9*-D374Y Does Not Affect the Proliferation of HepG2 Cells

Overexpression of exogenous genes can affect the normal proliferation of cells. To assess whether high expression of *PCSK9*-D374Y affects the proliferation of HepG2 cells, we examined the proliferation of *PCSK9*-D374Y overexpressed cells and control cells using CCK-8 assays and immunofluorescence. Our results demonstrated that *PCSK9*-D374Y does not affect the proliferation of HepG2 cells, as demonstrated by the standard curve and cell proliferation curve of HepG2 cells of different genotypes, according to CCK-8 assays (Figure S3). The proliferation cycle of HepG2 cells is 18-24 h. Both the control and *PCSK9*-D374Y cells peaked at 72 h, which indicates that *PCSK9*-D374Y overexpression has no significant effect on the proliferation of HepG2 cells.

To further investigate cell proliferation in a more accurate manner, we analyzed the cell proliferation level by counting EdU-positive and EdU-negative nuclei (Figure S4A and S4B). Fluorescence staining and statistical analysis demonstrate that *PCSK9*-D374Y overexpression has no significant effect on the proliferation of HepG2 cells. We also analyzed the expression level of proliferating cell nuclear antigen (PCNA), a marker for mitotic cells, in the nucleus and cytoplasm and did not find any difference in the expression of this protein in the cell lines (Figure S4C). This further confirms our conclusions at the molecular level.

### 3.4. *PCSK9*-D374Y Significantly Suppresses the Migration of HepG2 Cells

**Figure 5:**
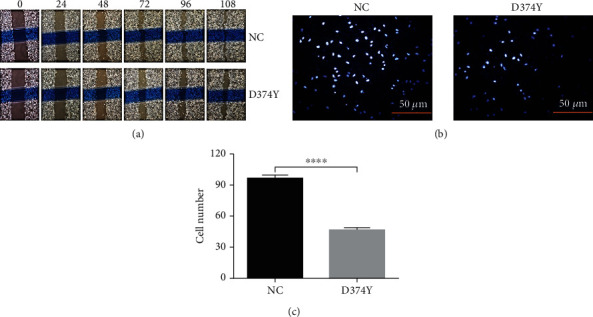
Effect of *PCSK9* overexpression on HepG2 cell migration. (a) Scratch wound healing assay in HepG2 cells. Photos were taken at 0 h, 24 h, 48 h, 72 h, 96 h, and 108 h at the same place of the scratch wound. At least three independent experiments were conducted with triplicate items. (b) Transwell migration assay of HepG2 cells. Images show representative results. Three random images were collected from each membrane of transwell. Data in (c) show the statistical analysis of (b). At least three independent experiments were conducted with triplicate items. The bar chart is shown as mean with SEM. ^∗∗∗∗^*P* < 0.001.

Cell migration is an important process by which cells exert normal biological functions. Few studies have investigated the effects of *PCSK9*-D374Y on the migration of hepatocytes. We performed scratch wound healing assays to identify the influence of *PCSK9* on the migration of HepG2 cells (Figure 5(a)). HepG2 control cells have a relatively weak migration capacity, and scratch wounds did not heal even after 108 h. Scratch wound healing is relatively slower in *PCSK9*-D374Y overexpression cells compared to control cells transfected with the vector, indicating that *PCSK9*-D374Y could suppress the migration of HepG2 cells. Transwell migration assays demonstrate the significant inhibitory effects of *PCSK9*-D374Y on the vertical migration of HepG2 cells (Figures 5(b) and 5(c)) (*P* < 0.0001, *t*-test).

**Figure 6:**
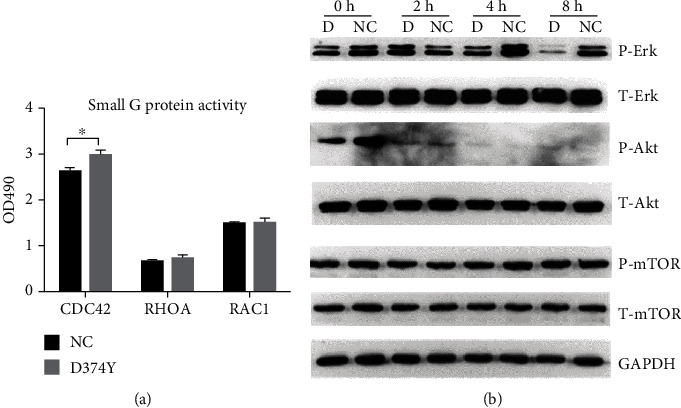
Analysis of signaling proteins related to cell migration. (a) Activity detection of three small G proteins (CDC42, RHOA, and RAC1) in HepG2 cells. (b) Detection of the phosphorylation levels of ERK, AKT, and mTOR proteins by western blot analysis. Phosphorylation levels of ERK, AKT, and mTOR proteins and the total protein levels in the control group and *PCSK9*-D374Y-transfected group were detected at 0 h, 2 h, 4 h, and 8 h after starvation treatment of HepG2 cells. At least three independent experiments were conducted. The bar chart is shown as mean with SEM. ^∗^*P* < 0.05.

Cell migration is closely related to the dynamic remodeling and signaling of cytoskeletal proteins. The small G proteins play key roles in the assembly of cytoskeleton proteins. However, when detecting the activities of three key small G proteins, we did not find any difference in the activities of RAC family small GTPase 1 (RAC1) and Ras homolog family member A (RHOA) between *PCSK9*-D374Y overexpression and control cells, while the activity of cell division cycle 42 (CDC42) was significantly upregulated in *PCSK9*-D374Y overexpression cells (Figure 6(a)) (*P* < 0.05). Moreover, the mRNA transcription of RHOA is downregulated (Figure 4(d)). We also examined the phosphorylation levels of migration-related proteins, including ERK, AKT, and mechanistic target of rapamycin kinase (mTOR) (Figure 6(b)). Fluctuations in the phosphorylation levels of ERK were observed in serum-starved control cells; phosphorylation levels of ERK decreased after the cells were subjected to serum starvation treatment for 2 h, peaked at 4 h, and declined again at 8 h. Compared with cells of *PCSK9*-D374Y overexpression, cells in the control group maintained relatively high phosphorylation levels of ERK throughout the assay, which exceeded that in the overexpression group at all time points (except at 2 h). In contrast, the phosphorylation levels of ERK in the *PCSK9*-D374Y overexpression group first increased and then decreased, reaching the lowest value at 8 h. This suggests that the ERK signaling pathway is involved in regulating the migration of HepG2 cells. These fluctuations are consistent with the low migration capability in the *PCSK9*-D374Y overexpression group. Under normal culture conditions, AKT phosphorylation levels were significantly higher in the control group than in the *PCSK9*-D374Y overexpression group (*P* = 0.0029, *t*-test); however, almost no phosphorylated AKT was detected during starvation treatment. This indicates that AKT might not be involved in regulating starvation-induced HepG2 cell migration. The mTOR phosphorylation level also shows no significant differences among the different groups.

## 4. Discussion

In this study, we achieved stable overexpression of the *PCSK9*-D374Y mutant in HepG2 cells, analyzed the proliferation, migration, and free cholesterol transport of *PCSK9*-D374Y overexpression cells, and investigated the effect of *PCSK9*-D374Y on the biological functions of hepatocytes. HepG2 cells with *PCSK9*-D374Y overexpression exhibited no change in overall cell proliferation compared with the control group; however, their free cholesterol transport capability and migration activities were significantly suppressed. *PCSK9*-D374Y can inhibit free cholesterol transport, and the cell migration ability is downregulated through the ERK signaling pathways.

The HepG2 cell line of *PCSK9*-D374Y can be used for studying the function of hepatocytes. This cell line has high *PCSK9*-D374Y transcription, translation, and protein secretion activities, and the abundance of LDLR proteins in the HepG2 cell membrane and cytoplasm was also validated in the overexpression cell line. The HepG2 cell line is a useful tool for investigating the synthesis, secretion, and molecular features of *PCSK9*, the binding capacity of *PCSK9* with LDLR, the ability of *PCSK9* to mediate LDLR degradation, and *PCSK9* mutants and their functions [25–27].

Previous studies have reported that the *PCSK9* expression level is correlated with the proliferation of hepatocytes and the metastasis of tumor cells [17, 28]. Therefore, we used the HepG2 cell line to explore the proliferation, migration, and free cholesterol transport of hepatocytes. In this study, our proliferation assay results demonstrate that *PCSK9* overexpression does not affect HepG2 hepatocyte proliferation. However, in their mouse liver injury models, Zaid et al. propose that *PCSK9* plays a key role in mouse liver repair and hepatocyte proliferation [17]. Repairing liver injuries in mice requires cholesterol; *PCSK9* gene deficiency decreases cholesterol levels in the microenvironment where the hepatocytes are located, thus inhibiting hepatocyte proliferation. However, the authors do not determine whether the *PCSK9* gene is directly involved in regulating hepatic cell proliferation, rather, that the effect of *PCSK9* on the cholesterol level in the microenvironment of liver tissue affects the repair of liver injury [17]. We also find out that overexpression of *PCSK9-*D374Y does not affect PCNA levels. As such, we further confirmed that *PCSK9-*D374Y overexpression does not affect hepatocyte proliferation. However, endogenous *PCSK9* can affect the proliferation results. In future studies, it is important to evaluate these phenomena using cells lacking endogenous *PCSK9* expression.

Sun et al. use mouse models with cancer cell metastasis to explore the effect of *PCSK9* on cancer cell metastasis and found that *PCSK9* deficiency inhibited the metastasis of cancer cells [28]. We demonstrate that *PCSK9* overexpression significantly inhibits the horizontal motion and vertical migration of HepG2 cells. The difference between our study and other studies can be explained by different cell models, suggesting that the effect of *PCSK9* on migration capacity is cell-specific. Sun et al. note that the effect of *PCSK9* on cell migration is based on the regulation of cholesterol levels; however, the authors do not elucidate the molecular mechanism by which *PCSK9* affects the migration capacity. Therefore, we speculated that the effect of *PCSK9* on cell migration can be achieved by regulating cholesterol metabolism in cells.

Studies have revealed that the distribution and content of free cholesterol on the cell membrane are important for the function of lipid rafts on the cell membrane and cellular signaling [29, 30]. Therefore, we examined cell migration-related signaling pathways based on the phenotype data and found that *PCSK9*-D374Y suppressed the migration capacity of HepG2 cells by downregulating the phosphorylation levels of ERK, confirming the results found in previous studies [31]. In the complete medium-containing serum, the phosphorylation levels of ERK of gene overexpressed cells were significantly lower than those of wild-type cells. This suggests that the kinase reserve of gene overexpression cell migration-related pathways was insufficient at the initial stage of the migration experiment. The activity of ERK signaling pathway has been proved to be involved in the regulation of cell migration in previous works. For example, in wound healing experiments, the migration of keratinocytes was enhanced by micrograft treatment through ERK activation [32]. Another research indicated that ovarian cancer cell migration was induced by betacellulin through EGFR-MEK/ERK signaling [33]. Thus, it may be involved in the regulation of cell migration dependent on ERK activity. During cell migration, serum-free culture conditions make cells that produce stress and migrate [34], which will consume the corresponding kinase. We also detected the efficiency of free cholesterol transport in HepG2 cells and found that compared with the cells in the control group, *PCSK9*-D374Y overexpression significantly suppressed the free cholesterol efflux rate in HepG2 cells without affecting the capacity of free cholesterol labeling. When the free cholesterol efflux is suppressed, cholesterol can accumulate in the inner surface of the cellular membrane, which may weaken the signal transduction related to lipid rafts. According to Pagler et al., increased cholesterol levels in the inner layer of the cellular membrane can lead to the tight binding of small G proteins to the inner surface of the cellular membrane, resulting in imbalanced RAC1/RHOA activity and decreasing cellular motility. In this study, we detected the activities and expression levels of small G proteins in HepG2 cells and did not find changes in their activities and expression, except for decreased RHOA expression. However, the activity of CDC42, another small G protein, significantly increased in the overexpression of cells. Further investigation is required to determine whether the abnormally increased CDC42 activity affects the ERK signaling pathways [35]. Additionally, while only the total expression volume of RAC1 and RHOA is detected in our study, the distribution of these two proteins on the cell membrane and lipid rafts also warrants further investigation.

In addition, we conducted animal experiments to prove the conclusions obtained in cell experiments. We adopted *PCSK9*-D374Y transgenic pigs. Hepatic sinus dilatation and unclear boundary of hepatic lobule can be observed on the liver of *PCSK9*-D374Y transgenic pigs. Furthermore, there are scattered lymphocyte infiltration on the liver lobule (Figure S5). Accordingly, we used *PCSK9*-KO mice to investigate the total cholesterol level in vivo. Results showed that serum levels of TC, TG, HDL-C, and LP (a) in *PCSK9*-KO mice were significantly downregulated when compared with WT mice (Figure S6). Meanwhile, quantitative proteomics results of primary hepatic cells isolated from *PCSK9*-KO mice also showed that many proteins involved in lipid transport were upregulated such as LDLR, APOA2, APOA4, NPC1, and ABCC2 (data not shown). These results indicated that *PCSK9* may negatively regulate cholesterol and lipid transport in vivo.

## 5. Conclusions

In summary, our results demonstrate that *PCSK9*-D374Y, a gain-of-function mutation, has no effect on HepG2 cell proliferation but exerts an inhibitory effect on cell migration and the cholesterol efflux capacity of HepG2 cells. It can interfere with the activities of small G proteins and the signaling of lipid raft-facilitated cell signaling pathways and is involved in the regulation of ERK signaling pathways.

## Data Availability

The original contributions presented in the study are included in the article/supplementary material; further inquiries can be directed to the corresponding authors.

## Abbreviations

AKT: Serine/threonine-specific protein kinase
CDC42: Cell division cycle 42
DMEM: Dulbecco's modified eagle medium
ERK: Extracellular signal-regulated kinase
FBS: Fetal bovine serum
HDL: High-density lipoprotein
LDL-C: Low-density lipoprotein cholesterol
LDLR: Low-density lipoprotein receptor
mTOR: Mechanistic target of rapamycin kinase
OD: Optical density
PCNA: Proliferating cell nuclear antigen
PCSK9: Proprotein convertase subtilisin-like/kexin type 9
RAC1: RAC family small GTPase 1
RHOA: Ras homolog family member A.

## Supplementary Materials

Supplementary Materials Figure S1: construction of the human *PCSK9*-D374Y overexpression vector. (A) Design of the *PCSK9*-D374Y overexpression vector. The core components include the MAR insulator, human APOE promoter, and *PCSK9*-D374Y mutant coding sequence in the pcDNA3.1 backbone plasmid. (B) Confirmation of the D374Y overexpression vector. Digestion products from *EcoRI* and *BmtI* digestion of expected sizes are confirmed: 1433 bp, 2467 bp, and 6577 bp. Lanes 1 and 3: the digestion products; lanes 2 and 4: circular plasmids; and lane 5: 1-kb DNA marker. Figure S2: verification of *PCSK9*-D374Y in the HepG2 stable cell line. (A) QPCR detection of *PCSK9*-D374Y mRNA levels in HepG2 cells expressing pcDNA3.1-*PCSK9*-D374Y compared with pcDNA3.1 vector-transfected cells. (B) Western blot analysis of *PCSK9* in HepG2 cells expressing *PCSK9*-D374Y compared with pcDNA3.1 vector-transfected cells. *GAPDH* is used as the internal reference control. (C) ELISA assays of *PCSK9*-D3794Y protein in HepG2 cell culture medium. NC and D374Y represent pcDNA3.1 control and pcDNA3.1-*PCSK9*-D374Y-transfected HepG2 cells, respectively. At least three independent experiments are conducted with triplicate items. The bar chart is shown as mean with SEM. ^∗∗∗∗^*P* < 0.001. Figure S3: effect of *PCSK9* D374Y overexpression on the growth of HepG2 cells. (A) The standard curve reflecting the correlation between the CCK-8 OD value and the number of HepG2 cells. (B) The growth curve of HepG2 cells detected by CCK-8. NC, control group, transfected with blank plasmid pcDNA3.1; D374Y, cells are transfected with D374Y overexpression plasmid. At least three independent experiments are conducted with triplicate items. The error bar represents mean with SEM. ^∗∗∗^*P* < 0.001. Figure S4: effect of *PCSK9* overexpression on the proliferation of HepG2 cells. (A, B) EdU cell proliferation assay. (A) EdU fluorescent staining results. The fluorescence signals indicate the nuclei; blue fluorescence indicates that the cells are in interphase and the red fluorescence represents cells in mitosis. (B) The bar chart for the results of EdU fluorescent staining. At least three independent experiments are conducted with triplicate items. The error bar represents mean with SEM. (C) Detection of the expression of *PCNA*, a marker for mitotic cells, in nuclear and cytoplasmic fractions. Histone 2A was used as the marker for the nuclear fraction and *β*-actin is used as the cytoplasm/total protein marker. NC: pcDNA3.1; D374Y: pcDNA3.1-*PCSK9*-D374Y; cytoplasm: cytoplasmic proteins; nucleus: nuclear proteins. Figure S5: H&E staining of the liver in *PCSK9*-D374Y and wild-type pigs. (A, B) H&E staining of liver in wild-type pigs. (C, D) H&E staining of liver in *PCSK9*-D374Y pigs. (B, D) The local expansion area in (A) or (C). Figure S6: biochemical detection of blood lipid in *PCSK9* knockout mice.
