# Supplementary material for: PCSK9-D374Y Suppresses Hepatocyte Migration through Downregulating Free Cholesterol Efflux Rate and Activity of Extracellular Signal-Regulated Kinase
Source: Anal Cell Pathol (Amst). 2023 Jan 9;2023:6985808. doi: 10.1155/2023/6985808 (PMC9842426; doi:10.1155/2023/6985808)
Supplement: Supplementary Materials — Figure S1: construction of the human PCSK9-D374Y overexpression vector. (A) Design of the PCSK9-D374Y overexpression vector. The core components include the MAR insulator, human APOE promoter, and PCSK9-D374Y mutant coding sequence in the pcDNA3.1 backbone plasmid. (B) Confirmation of the D374Y overexpression vector. Digestion products from EcoRI and BmtI digestion of expected sizes are confirmed: 1433 bp, 2467 bp, and 6577 bp. Lanes 1 and 3: the digestion products; lanes 2 and 4: circular plasmids; and lane 5: 1-kb DNA marker. Figure S2: verification of PCSK9-D374Y in the HepG2 stable cell line. (A) QPCR detection of PCSK9-D374Y mRNA levels in HepG2 cells expressing pcDNA3.1-PCSK9-D374Y compared with pcDNA3.1 vector-transfected cells. (B) Western blot analysis of PCSK9 in HepG2 cells expressing PCSK9-D374Y compared with pcDNA3.1 vector-transfected cells. GAPDH is used as the internal reference control. (C) ELISA assays of PCSK9-D3794Y protein in HepG2 cell culture medium. NC and D374Y represent pcDNA3.1 control and pcDNA3.1-PCSK9-D374Y-transfected HepG2 cells, respectively. At least three independent experiments are conducted with triplicate items. The bar chart is shown as mean with SEM. ∗∗∗∗P < 0.001. Figure S3: effect of PCSK9 D374Y overexpression on the growth of HepG2 cells. (A) The standard curve reflecting the correlation between the CCK-8 OD value and the number of HepG2 cells. (B) The growth curve of HepG2 cells detected by CCK-8. NC, control group, transfected with blank plasmid pcDNA3.1; D374Y, cells are transfected with D374Y overexpression plasmid. At least three independent experiments are conducted with triplicate items. The error bar represents mean with SEM. ∗∗∗P < 0.001. Figure S4: effect of PCSK9 overexpression on the proliferation of HepG2 cells. (A, B) EdU cell proliferation assay. (A) EdU fluorescent staining results. The fluorescence signals indicate the nuclei; blue fluorescence indicates that the cells are in interphase and the red fluoresc [file 6985808.f1.docx]

**Supplementary Figure Legends**

**Figure S1. Construction of the human *PCSK9*-D374Y overexpression vector.**

A) Design of the *PCSK9*-D374Y overexpression vector. The core components include the MAR insulator, human APOE promoter, and *PCSK9*-D374Y mutant coding sequence in the pcDNA3.1 backbone plasmid. B) Confirmation of the D374Y overexpression vector. Digestion products from *EcoRI* and *BmtI* digestion of expected sizes are confirmed: 1433 bp, 2467 bp, and 6577 bp. Lanes 1 and 3: the digestion products; lanes 2 and 4: circular plasmids; and lane 5: 1-kb DNA marker.

**Figure S2. Verification of *PCSK9*-D374Y in the HepG2 stable cell line.**

A) QPCR detection of *PCSK9*-D374Y mRNA levels in HepG2 cells expressing pcDNA3.1-*PCSK9*-D374Y compared with pcDNA3.1 vector-transfected cells. B) Western blot analysis of PCSK9 in HepG2 cells expressing *PCSK9*-D374Y compared with pcDNA3.1 vector-transfected cells. *GAPDH* is used as the internal reference control. C) ELISA assays of *PCSK9*-D3794Y protein in HepG2 cell culture medium. NC and D374Y represent pcDNA3.1 control and pcDNA3.1-*PCSK9*-D374Y-transfected HepG2 cells, respectively. At least three independent experiments are conducted with triplicate items. The bar chart is shown as Mean with SEM. ****, P<0.001.

**Figure S3. Effect of *PCSK9* D374Y over-expression on the growth of HepG2 cells.**

A) The standard curve reflecting the correlation between the CCK8 OD value and the number of HepG2 cells. B) The growth curve of HepG2 cells detected by CCK8. NC, control group, transfected with blank plasmid pcDNA3.1; D374Y, cells are transfected with D374Y over-expression plasmid. At least three independent experiments are conducted with triplicate items. The error bar represents Mean with SEM. ***, P<0.001.

**Figure S4. Effect of *PCSK9* over-expression on the proliferation of HepG2 cells.**

A, B) EdU cell proliferation assay. Figure A shows EdU fluorescent staining results. The fluorescence signals indicate the nuclei; blue fluorescence indicates that the cells are in interphase and the red fluorescence represents cells in mitosis. Figure B is the bar chart for the results of EdU fluorescent staining. At least three independent experiments are conducted with triplicate items. The error bar represents Mean with SEM. C) Detection of the expression of *PCNA*, a marker for mitotic cells, in nuclear and cytoplasmic fractions. Histone-2A was used as the marker for the nuclear fraction and β-actin is used as the cytoplasm/total protein marker. NC, pcDNA3.1; D374Y, pcDNA3.1- *PCSK9*-D374Y; cytoplasm, cytoplasmic proteins; nucleus, nuclear proteins.

**Figure S5. H&E staining of liver in *PCSK9*-D374Y and wild type pigs.**

A) & B) H&E staining of liver in wild type pigs. C) & D) H&E staining of liver in *PCSK9*-D374Y pigs. B) or D) showed the local expansion area in A) or C).

**Figure S6. Biochemical detection of blood lipid in *PCSK9* knockout mice.**

**
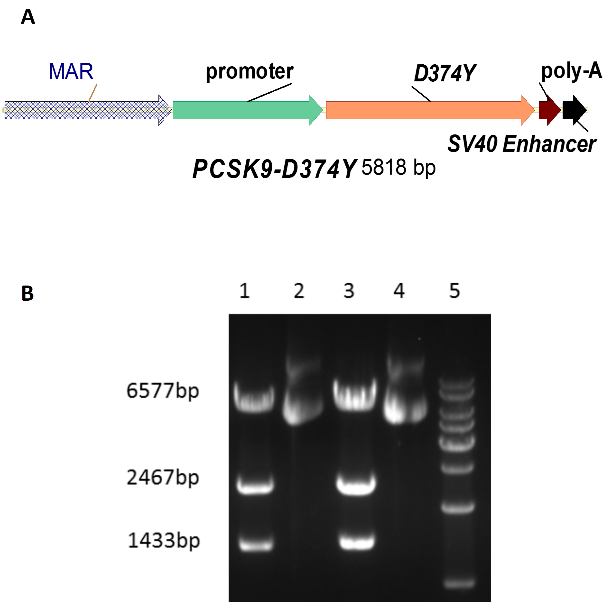
**

Figure S1. Construction of the human *PCSK9*-D374Y overexpression vector.


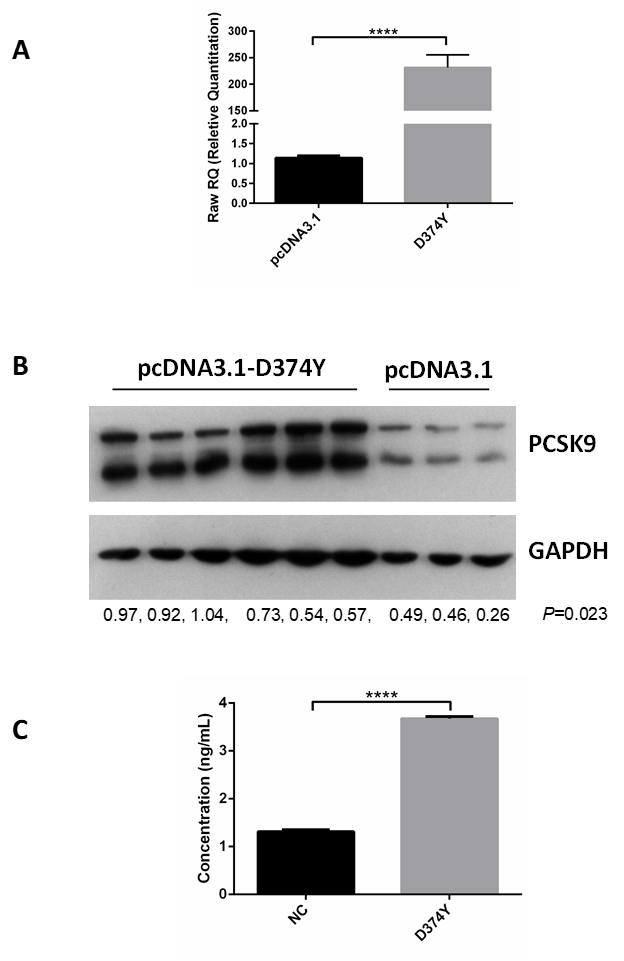


Figure S2 Verification of *PCSK9*-D374Y in the HepG2 stable cell line


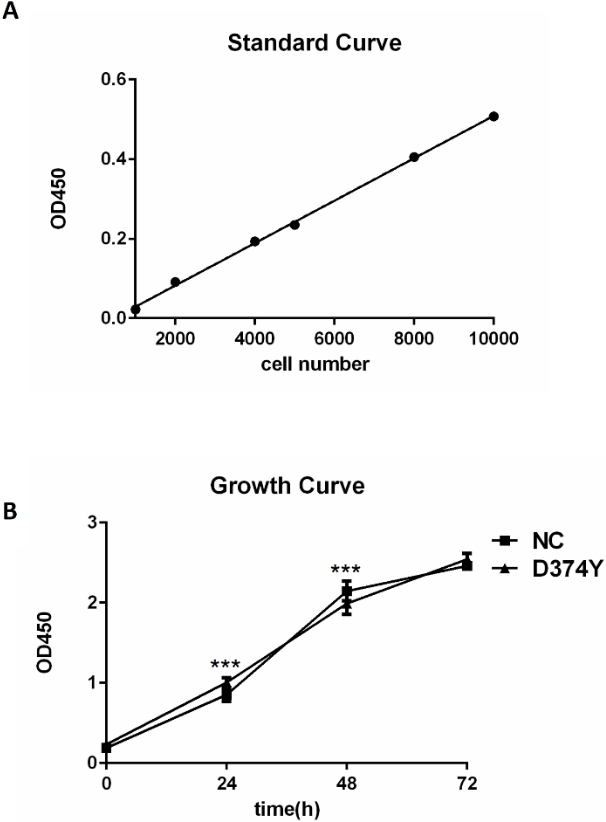


Figure S3. Effect of *PCSK9* D374Y over-expression on the growth of HepG2 cells


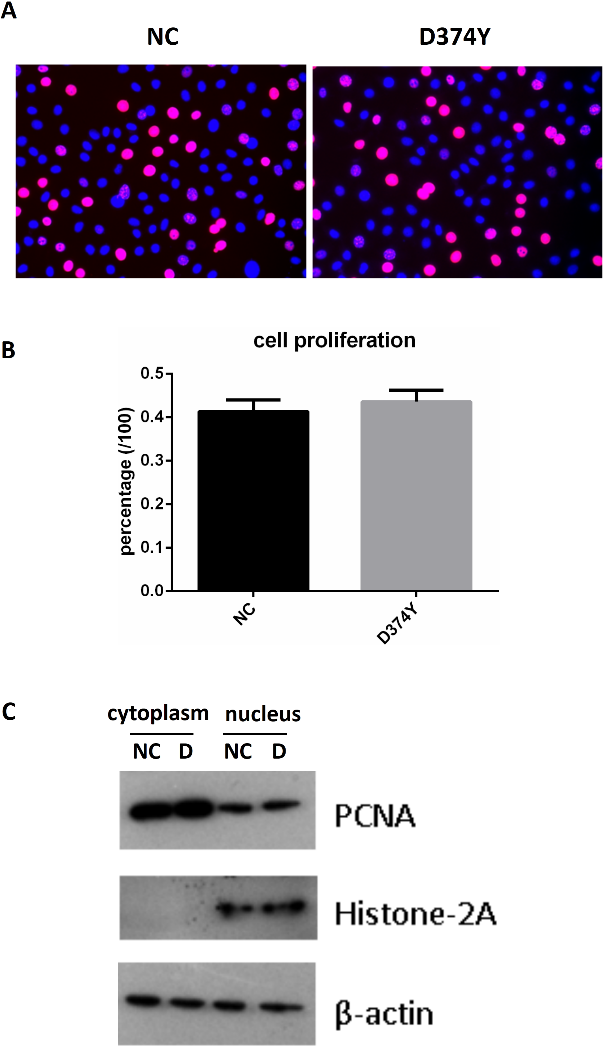


Figure S4. Effect of PCSK9 over-expression on the proliferation of HepG2 cells.

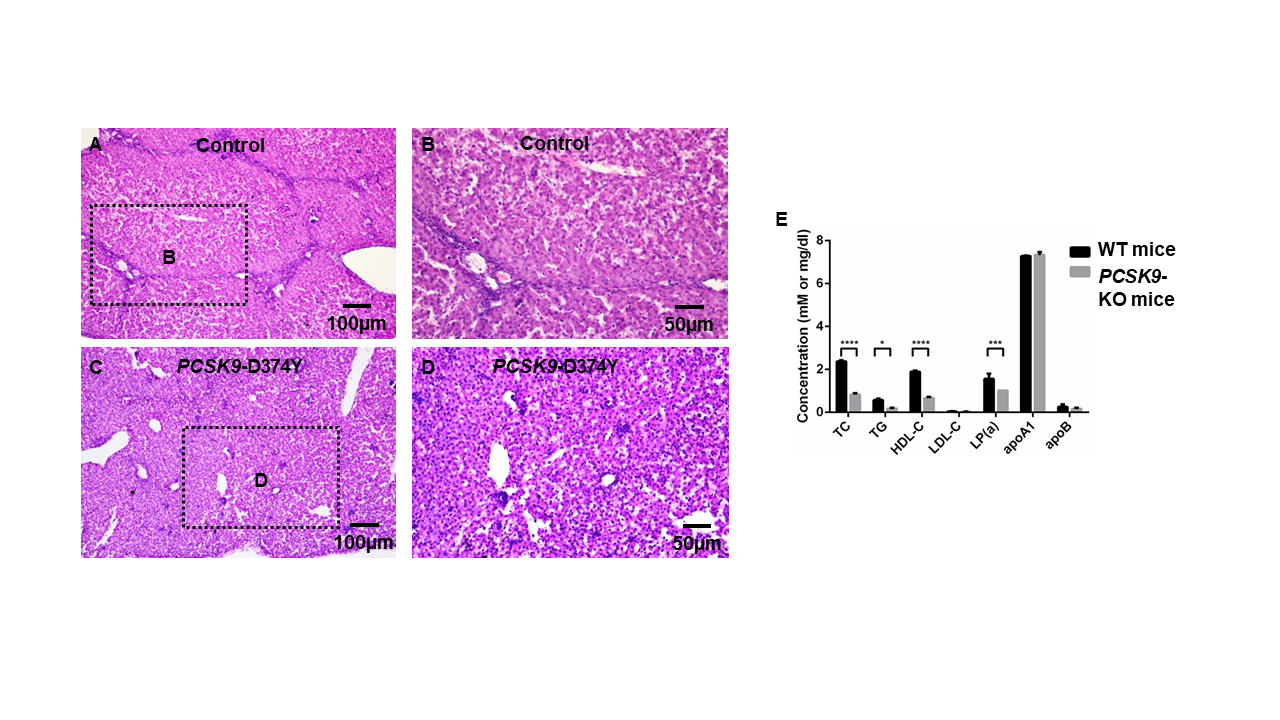
Figure S5. H&E staining of liver in *PCSK9*-D374Y and wild type pigs.

Figure S6. Biochemical detection of blood lipid in PCSK9 knockout mice.

**The cDNA sequences and mutation sites of PCSK9 are shown as below:**

Mutated codons are indicated in bold/red.

atgggcaccgtcagctccaggcggtcctggtggccgctgccactgctgctgctgctgctgctgctcctgggtcccgcgggcgcccgtgcgcaggaggacgaggacggcgactacgaggagctggtgctagccttgcgttccgaggaggacggcctggccgaagcacccgagcacggaaccacagccaccttccaccgctgcgccaaggatccgtggaggttgcctggcacctacgtggtggtgctgaaggaggagacccacctctcgcagtcagagcgcactgcccgccgcctgcaggcccaggctgcccgccggggatacctcaccaagatcctgcatgtcttccatggccttcttcctggcttcctggtgaagatgagtggcgacctgctggagctggccttgaagttgccccatgtcgactacatcgaggaggactcctctgtctttgcccagagcatcccgtggaacctggagcggattacccctccacggtaccgggcggatgaataccagccccccgacggaggcagcctggtggaggtgtatctcctagacaccagcatacagagtgaccaccgggaaatcgagggcagggtcatggtcaccgacttcgagaatgtgcccgaggaggacgggacccgcttccacagacaggccagcaagtgtgacagtcatggcacccacctggcaggggtggtcagcggccgggatgccggcgtggccaagggtgccagcatgcgcagcctgcgcgtgctcaactgccaagggaagggcacggttagcggcaccctcataggcctggagtttattcggaaaagccagctggtccagcctgtggggccactggtggtgctgctgcccctggcgggtgggtacagccgcgtcctcaacgccgcctgccagcgcctggcgagggctggggtcgtgctggtcaccgctgccggcaacttccgggacgatgcctgcctctactccccagcctcagctcccgaggtcatcacagttggggccaccaatgcccaagaccagccggtgaccctggggactttggggaccaactttggccgctgtgtggacctctttgccccaggggaggacatcattggtgcctccagc**tac**tgcagcacctgctttgtgtcacagagtgggacatcacaggctgctgcccacgtggctggcattgcagccatgatgctgtctgccgagccggagctcaccctggccgagttgaggcagagactgatccacttctctgccaaagatgtcatcaatgaggcctggttccctgaggaccagcgggtactgacccccaacctggtggccgccctgccccccagcacccatggggcaggttggcagctgttttgcaggactgtatggtcagcacactcggggcctacacggatggccacagccgtcgcccgctgcgccccagatgaggagctgctgagctgctccagtttctccaggagtgggaagcggcggggcgagcgcatggaggcccaagggggcaagctggtctgccgggcccacaacgcttttgggggtgagggtgtctacgccattgccaggtgctgcctgctaccccaggccaactgcagcgtccacacagctccaccagctgaggccagcatggggacccgtgtccactgccaccaacagggccacgtcctcacaggctgcagctcccactgggaggtggaggaccttggcacccacaagccgcctgtgctgaggccacgaggtcagcccaaccagtgcgtgggccacagggaggccagcatccacgcttcctgctgccatgccccaggtctggaatgcaaagtcaaggagcatggaatcccggcccctcaggagcaggtgaccgtggcctgcgaggagggctggaccctgactggctgcagtgccctccctgggacctcccacgtcctgggggcctacgccgtagacaacacgtgtgtagtcaggagccgggacgtcagcactacaggcagcaccagcgaaggggccgtgacagccgttgccatctgctgccggagccggcacctggcgcaggcctcccaggagctccagtga
